# Neutrophil Gelatinase-Associated Lipocalin Is Not Associated with Tacrolimus-Induced Acute Kidney Injury in Liver Transplant Patients Who Received Mycophenolate Mofetil with Delayed Introduction of Tacrolimus

**DOI:** 10.3390/ijms20123103

**Published:** 2019-06-25

**Authors:** Mio Fukuda, Kimitaka Suetsugu, Soichiro Tajima, Yurie Katsube, Hiroyuki Watanabe, Noboru Harada, Tomoharu Yoshizumi, Nobuaki Egashira, Masaki Mori, Satohiro Masuda

**Affiliations:** 1 Department of Pharmacy, Kyushu University Hospital, 3-1-1 Maidashi, Higashi-ku, Fukuoka 812-8582, Japan; miomio15@pharm.med.kyushu-u.ac.jp (M.F.); suetsugu@pharm.med.kyushu-u.ac.jp (K.S.); stajima@pharm.med.kyushu-u.ac.jp (S.T.); katsube.yurie.170@m.kyushu-u.ac.jp (Y.K.); hirwata@pharm.med.kyushu-u.ac.jp (H.W.); n-egashi@pharm.med.kyushu-u.ac.jp (N.E.); 2 Department of Clinical Pharmacology and Biopharmaceutics, Graduate School of Pharmaceutical Sciences, Kyushu University, 3-1-1 Maidashi, Higashi-ku, Fukuoka 812-8582, Japan; 3 Department of Surgery and Science, Graduate School of Medical Sciences, Kyushu University, 3-1-1 Maidashi, Higashi-ku, Fukuoka 812-8582, Japan; nharada@surg2.med.kyushu-u.ac.jp (N.H.); tomyoshi@surg2.med.kyushu-u.ac.jp (T.Y.); m_mori@surg2.med.kyushu-u.ac.jp (M.M.)

**Keywords:** tacrolimus, acute kidney injury, biomarker, liver transplantation, neutrophil gelatinase-associated lipocalin

## Abstract

Tacrolimus is widely used as an immunosuppressant in liver transplantation, and tacrolimus-induced acute kidney injury (AKI) is a serious complication. The urinary neutrophil gelatinase-associated lipocalin (NGAL) level has been linked to tacrolimus-induced AKI in patients starting tacrolimus treatment the morning after liver transplantation. Here we tested this association using a different immunosuppression protocol: Mycophenolate mofetil administration beginning on Postoperative Day 1 and tacrolimus administration beginning on Postoperative Day 2 or 3. Urine samples were collected from 26 living donor liver transplant recipients before (Postoperative Day 1) and after (Postoperative Day 7 or 14) tacrolimus administration. NGAL levels were measured via enzyme-linked immunosorbent assays, as were those of three additional urinary biomarkers for kidney diseases: Monocyte chemotactic protein-1 (MCP-1), liver-type fatty acid-binding protein (L-FABP), and human epididymis secretory protein 4 (HE4). HE4 levels after tacrolimus administration were significantly higher in patients who developed AKI (*n* = 6) than in those who did not (*n* = 20), whereas NGAL, MCP-1, and L-FABP levels did not differ significantly before or after tacrolimus administration. These findings indicate that NGAL may not be a universal biomarker of AKI in tacrolimus-treated liver transplant recipients. To reduce the likelihood of tacrolimus-induced AKI, our immunosuppression protocol is recommended.

## 1. Introduction

Tacrolimus, a calcineurin inhibitor (CNI), is widely used as an immunosuppressant in patients undergoing liver transplantation. Maintenance of its blood concentration within its narrow therapeutic range (5–15 ng/mL) is important for preventing adverse reactions such as nephrotoxicity and neurotoxicity [1].

CNI-induced acute kidney injury (AKI) is a frequent and serious complication of liver transplantation. AKI can lead to chronic kidney disease and has been associated with high mortality rates in liver transplant recipients [2,3]. The Kidney Disease Improving Global Outcomes (KDIGO) foundation defines AKI as any of the following: (1) An increase in the serum creatinine (SCr) level of 0.3 mg/dL within 48 h; (2) an increase in the SCr level of ≥1.5 times the baseline level within or presumably within a seven-day period; and (3) a urine volume of 0.5 mL/kg/h for 6 h.

Whether SCr is a reliable indicator of AKI is unclear for the following reasons. First, SCr levels reflect changes in the glomerular filtration rate (GFR) [4], which is a nonspecific measure of proximal tubular injury that is usually apparent only after significant kidney damage [5]. Second, non-renal factors also affect SCr levels; these include age, sex, body weight, muscle mass, total body volume, and protein intake [4,6,7]. Third, estimated GFRs (eGFRs) based on SCr levels are imprecise when determined before or after liver transplantation (especially in patients with end-stage liver disease) or perioperatively for assessment of dynamic renal function [8]. Additionally, although the KDIGO diagnostic criteria are the best indicators of AKI in intensive care units, not all liver transplant recipients undergo KDIGO criteria-based evaluations. Therefore, there is a need for more sensitive and specific biomarkers of AKI.

Neutrophil gelatinase-associated lipocalin (NGAL) detects AKI with higher sensitivity and specificity than does SCr. As such, it aids recognition of AKI in the emergency room [9] and after cardiac surgery and liver transplantation [10,11]. A recent study found that high urinary levels of NGAL predicted tacrolimus-induced AKI in liver transplant recipients in whom tacrolimus administration was initiated the morning after surgery (Postoperative Day 1) [12]. However, the generalization of this result is unclear because protocols for administering immunosuppressants differ among institutions. Therefore, validation of this finding requires further investigation.

Here, we determined whether the urinary level of NGAL predicted or detected tacrolimus-induced AKI in liver transplant recipients who received mycophenolate mofetil (MMF) the morning after surgery (Postoperative Day 1) and tacrolimus within three days after surgery.

## 2. Results

### 2.1. Patient Characteristics

Our study comprised 26 patients (6 men and 20 women) who underwent living donor liver transplantation (LDLT) at our hospital. All patients received MMF the day after surgery and tacrolimus within three days of surgery. Among these, six (23.1%) developed AKI during the 14-day postoperative period (the tacrolimus (TAC)–AKI group) and 20 did not (the non-AKI group) (Table 1). Graft volume (GV) (*p* = 0.006) and GV per standard liver volume (GV/SLV) (*p =* 0.037) were significantly higher in the non-AKI group than in the TAC–AKI group (Table 1). GV per body weight (GV/BW) did not differ significantly between the groups nor did age, sex, body weight, Child–Pugh score, Model for End-stage Liver Disease score, preoperative SCr level, preoperative blood urea nitrogen level, preoperative eGFR, postoperative blood tacrolimus level, or total postoperative dose of tacrolimus. Time course of serum creatinine levels in all patients with TAC–AKI and in those without AKI are described in the Supplementary Materials (Supplementary Figure S1).

### 2.2. Diagnostic and Predictive Ability of Urinary Biomarkers

**Figure 1:**
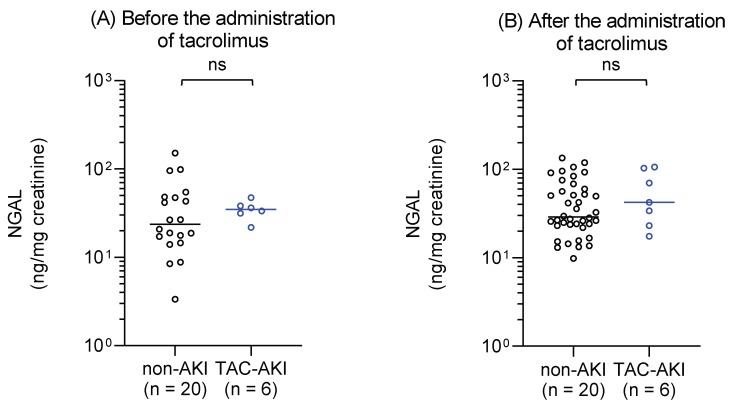
Urinary levels of neutrophil gelatinase-associated lipocalin (NGAL) in the non-AKI and TAC–AKI groups before and after the administration of tacrolimus. (**A**) Urinary samples were collected on Postoperative Day 1 immediately before the administration of tacrolimus. There were 20 measurements (20 subjects) in the non-AKI group and six measurements (six subjects) in the TAC–AKI group. (**B**) Urinary samples were collected during tacrolimus therapy (either Postoperative Day 7 or 14). There were 40 measurements in the non-AKI group (20 subjects) and seven measurements in the TAC–AKI group (six subjects).

Urinary NGAL levels were measured in urine samples collected from patients in the TAC–AKI and non-AKI groups immediately before tacrolimus administration on Postoperative Day 1 (Figure 1A) and during tacrolimus treatment (Postoperative Day 7 or 14) (Figure 1B). They did not differ significantly between the groups before or after tacrolimus administration.

**Figure 2:**
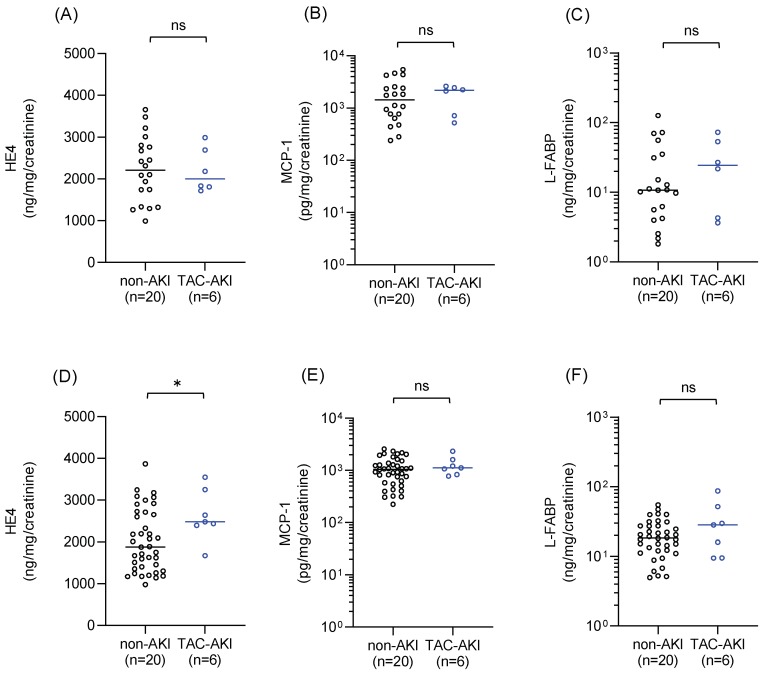
Urinary levels of human epididymis secretory protein 4 (HE4). (**A**) Monocyte chemotactic protein-1 (MCP-1) (**B**), and liver-type fatty acid-binding protein (L-FABP) (**C**) in the non-AKI group and TAC–AKI group immediately before tacrolimus administration on Postoperative Day 1. There were 20 measurements in the non-AKI group (20 subjects) and six measurements in the TAC–AKI group (six subjects). Urinary levels of HE4 (**D**), MCP-1 (**E**), and L-FABP (**F**) in the non-AKI and TAC–AKI groups during tacrolimus therapy (either Postoperative Day 7 or 14). There were 40 measurements in the non-AKI group (20 subjects) and seven measurements in the TAC–AKI group (six subjects).

The levels of three additional urinary biomarkers were measured in urine samples collected immediately before administration of tacrolimus on Postoperative Day 1 (Figure 2A–C) and during tacrolimus treatment (either Postoperative Day 7 or 14) (Figure 2D–F). Urinary levels of monocyte chemoattractant protein 1 (MCP-1) and liver-type fatty acid-binding protein (L-FABP) did not differ between the TAC–AKI and non-AKI groups before or after tacrolimus administration. In contrast, the urinary level of human epididymis secretory protein 4 (HE4) was significantly higher in the TAC–AKI versus non-AKI group during tacrolimus treatment (*p* = 0.042). 

NGAL levels were normalized to urinary creatinine levels and plotted on a logarithmic y-axis. Statistical analyses were performed using the Mann–Whitney *U* test. AKI, acute kidney injury; NGAL, neutrophil gelatinase-associated lipocalin; ns, not significant; TAC, tacrolimus. 

MCP-1 and L-FABP levels were normalized to urinary creatinine levels and plotted on a logarithmic y-axis. HE4 levels were normalized to urinary creatinine levels and plotted on the y-axis as real numbers. Statistical analyses were performed using the Mann–Whitney U test. * *p* < 0.05. AKI, acute kidney injury; HE4, human epididymis secretory protein 4; L-FABP, liver-type fatty acid-binding protein; MCP-1, monocyte chemoattractant protein 1; ns, not significant; TAC, tacrolimus.

## 3. Discussion

AKI is a common and significant complication of liver transplantation [3,13], and various biomarkers for AKI have been identified. Among them, NGAL accurately detects AKI in patients treated in emergency rooms [9] and patients who underwent cardiac surgery [14,15] or liver transplantation [10,11]. In addition, a previous study reported a significant association between high urinary levels of NGAL and tacrolimus-induced AKI in living-donor liver transplant recipients [12]. Here, we examined urinary levels of NGAL in patients who underwent LDLT at Kyushu University Hospital. No association between urinary NGAL levels and tacrolimus-induced AKI was found either before or after tacrolimus administration. 

NGAL, a 25-kDa protein, was originally isolated from human neutrophils [16] and is expressed at very low concentrations in the bone marrow and organs such as the trachea, kidney, lungs, and stomach [17]. In humans, NGAL is highly overexpressed after renal ischemic injury owing to enhanced gene transcription; urinary NGAL levels also increased soon after ischemic renal injury in mouse and rat models [18]. When complexed with a siderophore and iron, NGAL upregulates heme oxygenase-1 to preserve proximal tubules and prevent cell death [19]. It may be a useful marker for renal ischemic injury occurring after cardiac surgery [14,15] or liver transplantation [10,11], and for acute tubular injury (e.g., cisplatin-induced AKI [20] and contrast-induced nephropathy [21]). 

Complications caused by CNIs include structural damage to the straight segment of the proximal tubule [22], renal vasoconstriction mediated by the renal sympathetic nervous system [23], and interstitial fibrosis [24]. Interstitial fibrosis due to tacrolimus-induced neurotoxicity has been linked to overexpression of transforming growth factor beta 1 [10,11]. Previous reports correlate NGAL and other proteins expressed in both the proximal and distal tubules of the kidney with renal vasoconstriction and interstitial fibrosis caused by tacrolimus-induced nephrotoxicity [10,11]. 

**Figure 3:**
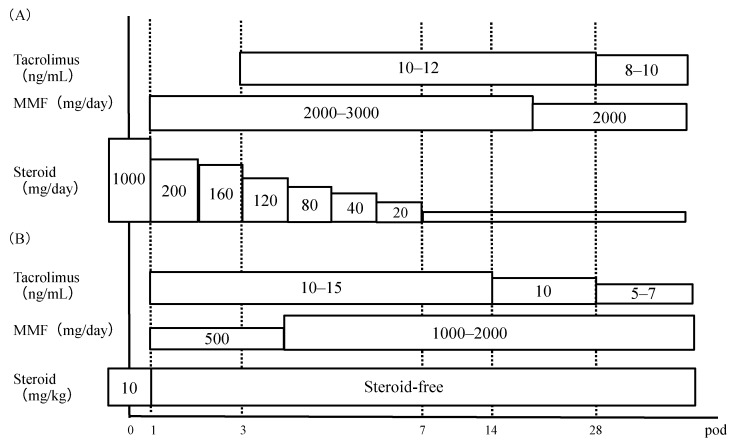
Immunosuppression protocols. (**A**) Kyushu University Hospital. (**B**) Kyoto University Hospital. MMF, mycophenolate mofetil; pod, postoperative day.

Unlike a previous study [12], we found no association between urinary NGAL levels and tacrolimus-induced AKI. This difference may reflect differences in the postoperative immunosuppression protocols used by Kyushu University Hospital (the site of our study) and Kyoto University Hospital (the site of the previous study) (Figure 3) [12]. At Kyoto University Hospital, the target trough blood concentrations of tacrolimus during the first two weeks after surgery were higher than those at our hospital. This is an important consideration because high postoperative trough concentrations of tacrolimus can cause nephrotoxicity and neurotoxicity [25]. To mention another difference, liver transplant recipients at Kyoto University Hospital received tacrolimus beginning the morning after surgery, whereas at our hospital, tacrolimus administration was delayed (second or third day after surgery) for renal protection. In addition to tacrolimus, the patients at our hospital also received MMF (2–3 g daily) beginning the day after surgery.

In many end-stage liver failure cases, renal arterioles contract and the contractions are exacerbated by CNI administration immediately after transplantation. In a previous study, the combination of MMF and a CNI prevented acute cellular rejection, and MMF mitigated the side effects of CNIs after kidney, heart, and liver transplantation [26]. Some reports advocate the use of immunosuppression therapies with low nephrotoxic potentials to reduce the risk of AKI after LDLT, [25] and delayed administration of tacrolimus to preserve renal function [27]. Based on these findings and recommendations, our hospital administered MMF and corticosteroids the day after transplantation and CNIs two–three days after transplantation for immunosuppression. Actually, the percentage of LDLT patients who developed AKI after tacrolimus administration was lower in our study (23.1%) than in the previous study at Kyoto Hospital (64.5%) [12]. An application of our protocol may reduce the incidence of tacrolimus-induced AKI after LDLT. 

Risk factors for AKI after LDLT include graft size, donor age [28], and preoperative levels [29]. Preoperative SCr level did not differ significantly between the non-AKI and TAC–AKI groups in our study. GV and GV/SLV were significantly higher in the TAC–AKI group than in the non-AKI group, whereas GV/BW did not differ significantly between the groups. These findings suggest that graft size is unrelated to tacrolimus-induced AKI. The tacrolimus-induced AKI in our study was likely caused by a combination of factors, including intra-operative renal injury, postoperative complications, and side effects of the medications, especially tacrolimus. Through blood concentrations of tacrolimus may also have contributed; although not significant, they tended to be higher in the TAC–AKI group. A low eGFR was not a factor because patients with a preoperative eGFR <60 mL/min/1.73 m^2^ were excluded. 

As a novel discovery, urinary levels of HE4 were significantly higher in the TAC–AKI group than in the non-AKI group during post-transplantation tacrolimus treatment. HE4 is a secreted protein encoded by the *WFDC2* gene, which is located on chromosome 20q12-131 [30]. It reproducibly identifies epithelial ovarian cancers, endometrial carcinomas, and lung malignancies, [6,7,8] and is a potential biomarker for renal fibrosis, cystic fibrosis, lupus nephritis, and chronic kidney disease in systemic lupus erythematosus [31,32]. The physiological and pathological functions of HE4 are not completely understood. HE4 is upregulated in fibrotic kidneys where it promotes renal fibrosis by suppressing the activity of serine proteases and matrix metalloproteinases and, consequently, the degradation of type I collagen [31]. In patients with kidney allografts, elevated HE4 levels may induce renal fibrosis [33] and increase the severity of tubulointerstitial fibrosis and tubular cell damage [34]. The increase in HE4 urinary levels, but not NGAL urinary levels, in the TAC–AKI group suggests that tacrolimus induces AKI by triggering the release of cytokines and chemokines, which exacerbate tubular injury. HE4 may also contribute to interstitial fibrosis by stimulating myoblast differentiation.

This study suggests that urinary levels of NGAL do not predict or detect tacrolimus-induced AKI in liver transplant patients who received MMF and delayed introduction of tacrolimus. The patients in our study had less frequent nephrotoxicity reactions than those who began tacrolimus treatment the morning after liver transplantation [12]. It is possible that tacrolimus induces AKI by different mechanisms depending on the postoperative immunosuppression treatment. Delaying tacrolimus administration after transplantation may reduce the incidence of AKI. Additionally, more sensitive and novel biomarkers for identifying tacrolimus-induced AKI during the postoperative immunosuppression treatment period regardless of postoperative immunosuppressive treatment are needed. Further analysis with a larger sample size is required to assess the accuracy of the present results.

## 4. Materials and Methods 

### 4.1. Patients

**Figure 4:**
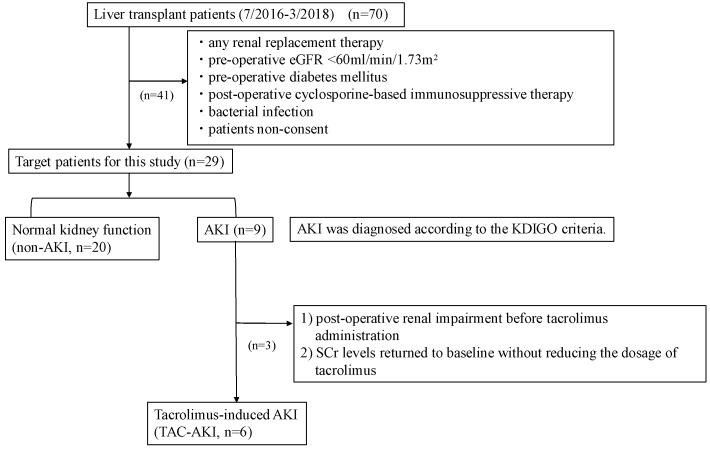
Diagnostic algorithm of patient selection. A total of 70 patients (29 men and 41 women; age, >18 years) who underwent living donor liver transplantation at Kyushu University Hospital between July 2016 and March 2018 were enrolled in the study after obtaining written informed consent. Patients with renal impairment due to septic ischemia, antibiotics, hepatorenal syndrome, or other causes and patients who had received renal replacement therapy were excluded. Additionally excluded were patients with renal impairment despite low tacrolimus levels, patients whose SCr levels were unaffected by changes in the tacrolimus dosage, patients with perioperative renal impairment before postoperative tacrolimus-based immunosuppression treatment, and patients with a preoperative eGFR (<60 mL/min/1.73 m^2^) or diabetes mellitus. Ultimately, 20 patients without AKI (control) and 6 patients with tacrolimus-induced AKI were included in the analysis. AKI, acute kidney injury; eGFR, estimated glomerular filtration rate; KDIGO, Kidney Disease Improving Global Outcomes; SCr, serum creatinine.

A total of 70 patients (29 men and 41 women; age, >18 years) underwent LDLT at Kyushu University Hospital between July 2016 and March 2018 (Figure 4). Patients with renal impairment due to septic ischemia, antibiotics, hepatorenal syndrome, or other causes, or who received renal replacement therapy, were excluded from the study. Also excluded were patients with renal impairment despite low tacrolimus levels, patients whose SCr levels were unaffected by changes in the tacrolimus dosage, patients with perioperative renal impairment before postoperative administration of immunosuppressants, and patients with a preoperative eGFR (<60 mL/min/1.73 m^2^) or diabetes mellitus. Ultimately, our study comprised 26 liver transplant recipients (6 men and 20 women).

Tacrolimus-induced AKI was diagnosed according to the KDIGO diagnostic criteria. The clinical, treatment, and laboratory data for all patients were obtained from electronic medical records. The preoperative eGFR for Japanese patients was calculated as follows:

eGFR=194 ×age −0.287 ×SCr −1.094 (×0.739 for women).

This study was conducted in accordance with the Declaration of Helsinki and its amendments, and was approved by the Ethics Committee of Kyushu University Graduate School and Faculty of Medicine (Approved number: 588-04, 26 December 2016). All patients provided written informed consent.

### 4.2. Urine Samples

Spot urine samples were collected immediately before the administration of tacrolimus on Postoperative Day 1 and on Postoperative Days 7 and 14. All urine samples were stored at −80 °C with protease inhibitor cocktail tablets (Complete Mini; Roche Diagnostics, Mannheim, Germany). Urinary creatinine levels were measured according to the Jaffe reaction using a Lab Assay Creatinine kit (Wako Pure Chemical Industries Ltd., Osaka, Japan). Urinary biomarker levels were measured using ELISA kits purchased from R&D Systems (Minneapolis, MN) (NGAL and HE4), CMIC Co., Ltd. (Tokyo, Japan) (L-FABP), and Abcam (Cambridge, UK) (MCP-1). Biomarker levels were normalized to urinary creatinine levels to adjust for changes in urine concentration.

### 4.3. Immunosuppression Protocol

Tacrolimus, steroids, and MMF were administered as part of the initial triple immunosuppression regimen or were introduced during the follow-up as a maintenance regimen. In all liver transplant patients, MMF (2000–3000 mg/day) was initiated the morning after surgery (Postoperative Day 1) and tacrolimus (2–4 mg/day) was started within 3 days after surgery. The tacrolimus dose was adjusted according to the clinical needs of the patient; the target whole-blood trough level was around 10–12 ng/mL for the first month after transplantation and 5–8 ng/mL thereafter. Intravenous methylprednisolone (1000 mg) was administered immediately after portal vein reperfusion and hepatic artery reperfusion, and tapered from 200 mg/day to 20 mg/day within about 6 days. It was continued at 20 mg/day for 1 month after transplantation and tapered to 0–5 mg/day after about 3–4 months (Figure 3). The blood concentration of tacrolimus was measured using a chemiluminescent enzyme immunoassay (ARCHITECT; Abbott Laboratories, Lake Bluff, IL, USA). 

### 4.4. Statistical Analyses

All statistical analyses were performed using Prism version 8 software (GraphPad Software, Inc., San Diego, CA). The Mann–Whitney U-test was used to compare differences between urinary biomarker levels in patients with/without AKI. A *p* value <0.05 was considered statistically significant.

## 5. Conclusions

There was no association between urinary NGAL levels and tacrolimus-induced AKI in our study. Hence, the urinary level of NGAL may not be a universal predictor of AKI in liver transplant recipients receiving tacrolimus. To reduce the likelihood of tacrolimus-induced AKI, we recommend administering tacrolimus 2–3 days after surgery, compensating immunosuppressive treatment with MMF from the morning after the surgery.

## Figures and Tables

**Table 1:** Patient characteristics.

Characteristic	Non-AKI (*n* = 20)	TAC–AKI (*n* = 6)	*p*-Value
Age (years)	53.6 ± 11.3	47.4 ± 6.63	0.046
Sex (male/female)	6/14	0/6	NS
Preoperative body weight (kg)	60.0 ± 10.68	58.0 ± 10.13	NS
Preoperative SCr (mg/dL)	0.57 ± 0.15	0.57 ± 0.10	NS
Preoperative BUN (mg/dL)	10.4 ± 4.4	13.8 ± 8.7	NS
Preoperative eGFR (mL/min/1.73 m^2^)	103.9 ± 30.2	90.8 ± 19.4	NS
MELD score	15.5 ± 6.75	15.0 ± 6.45	NS
Child–Pugh score	10.2 ± 1.9	10.5 ± 2.4	NS
GV (g)	525.0 ± 106.6	416.2 ± 47.2	0.006
GV/SLV (%)	45.5 ± 7.4	37.2 ± 5.2	0.037
GV/preoperative body weight (%)	0.88 ± 0.15	0.73 ± 0.14	NS
Primary disease (*n*)			NS
Primary biliary cirrhosis	3	5	
Hepatitis C virus-related liver cancer	5	1	
Hepatitis B virus	3	0	
Alcoholic cirrhosis	5	0	
Other	4	0	
Total tacrolimus dose between PODs 1 and 14 (mg)	59.0 ± 20.6	50.64 ± 20.75	NS
Blood tacrolimus between PODs 1 and 14 (ng/mL)	10.71 ± 1.9	12.08 ± 1.00	NS
ABO blood group match			NS
Identical/Compatible/Incompatible	11/3/6	4/1/1	

The results are given as mean ± standard deviation. Statistical analysis was performed using the Mann–Whitney *U* test and chi-square test. Abbreviations: AKI, acute kidney injury; BUN, blood urea nitrogen; eGFR, estimated glomerular filtration rate; GV, graft volume; MELD, Model for End-stage Liver Disease; NS, not significant; POD, postoperative day; SLV, standard liver volume; SCr, serum creatinine; TAC, tacrolimus.

## Supplementary Materials

Supplementary materials can be found at https://www.mdpi.com/1422-0067/20/12/3103/s1.

## References

[1] Masuda S., Inui K. (2006). An up-date review on individualized dosage adjustment of calcineurin inhibitors in organ transplant patients. Pharmacol. Ther..

[2] Lima E.Q., Zanetta D.M., Castro I., Massarollo P.C., Mies S., Machado M.M., Yu L. (2003). Risk factors for development of acute renal failure after liver transplantation. Ren. Fail..

[3] O’Riordan A., Wong V., McQuillan R., McCormick P.A., Hegarty J.E., Watson A.J. (2007). Acute renal disease, as defined by the RIFLE criteria, post-liver transplantation. Am. J. Transplant..

[4] Sieber M., Hoffmann D., Adler M., Vaidya V.S., Clement M., Bonventre J.V., Zidek N., Rached E., Amberg A., Callanan J.J. (2009). Comparative analysis of novel noninvasive renal biomarkers and metabonomic changes in a rat model of gentamicin nephrotoxicity. Toxicol. Sci..

[5] Sherman D.S., Fish D.N., Teitelbaum I. (2003). Assessing renal function in cirrhotic patients: Problems and pitfalls. Am. J. Kidney Dis..

[6] Bonventre J.V., Vaidya V.S., Schmouder R., Feig P., Dieterle F. (2010). Next-generation biomarkers for detecting kidney toxicity. Nat. Biotechnol..

[7] Vaidya V.S., Waikar S.S., Ferguson M.A., Collings F.B., Sunderland K., Gioules C., Bradwin G., Matsouaka R., Betensky R.A., Curhan G.C. (2008). Urinary biomarkers for sensitive and specific detection of acute kidney injury in humans. Clin. Transl. Sci..

[8] Charlton M.R., Wall W.J., Ojo A.O., Gines P., Textor S., Shihab F.S., Marotta P., Cantarovich M., Eason J.D., Wiesner R.H. (2009). International Liver Transplantation Society Expert Panel. Report of the first international liver transplantation society expert panel consensus conference on renal insufficiency in liver transplantation. Liver Transpl..

[9] Nickolas T.L., O’Rourke M.J., Yang J., Sise M.E., Canetta P.A., Barasch N., Buchen C., Khan F., Mori K., Giglio J. (2008). Sensitivity and specificity of a single emergency department measurement of urinary neutrophil gelatinase-associated lipocalin for diagnosing acute kidney injury. Ann. Intern. Med..

[10] Niemann C.U., Walia A., Waldman J., Davio M., Roberts J.P., Hirose R., Feiner J. (2009). Acute kidney injury during liver transplantation as determined by neutrophil gelatinase-associated lipocalin. Liver Transpl..

[11] Wagener G., Minhaz M., Mattis F.A., Kim M., Emond J.C., Lee H.T. (2011). Urinary neutrophil gelatinase-associated lipocalin as a marker of acute kidney injury after orthotopic liver transplantation. Nephrol. Dial. Transplant..

[12] Tsuchimoto A., Shinke H., Uesugi M., Kikuchi M., Hashimoto E., Sato T., Ogura Y., Hata K., Fujimoto Y., Kaido T. (2014). Urinary neutrophil gelatinase-associated lipocalin: A useful biomarker for tacrolimus-induced acute kidney injury in liver transplant patients. PLoS ONE.

[13] Hilmi I.A., Damian D., Al-Khafaji A., Planinsic R., Boucek C., Sakai T., Chang C.C., Kellum J.A. (2015). Acute kidney injury following orthotopic liver transplantation: Incidence, risk factors, and effects on patient and graft outcomes. Br. J. Anaesth..

[14] Bennett M., Dent C.L., Ma Q., Dastrala S., Grenier F., Workman R., Syed H., Ali S., Barasch J., Devarajan P. (2008). Urine NGAL predicts severity of acute kidney injury after cardiac surgery: A prospective study. Clin. J. Am. Soc. Nephrol..

[15] Mishra J., Dent C., Tarabishi R., Mitsnefes M.M., Ma Q., Kelly C., Ruff S.M., Zahedi K., Shao M., Bean J. (2005). Neutrophil gelatinase-associated lipocalin (NGAL) as a biomarker for acute renal injury after cardiac surgery. Lancet.

[16] Kjeldsen L., Johnsen A.H., Sengelov H., Borregaard N. (1993). Isolation and primary structure of NGAL, a novel protein associated with human neutrophil gelatinase. J. Biol. Chem..

[17] Cowland J.B., Borregaard N. (1997). Molecular characterization and pattern of tissue expression of the gene for neutrophil gelatinase-associated lipocalin from humans. Genomics.

[18] Mishra J. (2003). Identification of neutrophil gelatinase-associated lipocalin as a novel early urinary biomarker for ischemic renal injury. J. Am. Soc. Nephrol..

[19] Mori K., Lee H.T., Rapoport D., Drexler I.R., Foster K., Yang J., Schmidt-Ott K.M., Chen X., Li J.Y., Weiss S. (2005). Endocytic delivery of lipocalin-siderophore-iron complex rescues the kidney from ischemia-reperfusion injury. J. Clin. Invest..

[20] Gaspari F., Cravedi P., Mandala M., Perico N., de Leon F.R., Stucchi N., Ferrari S., Labianca R., Remuzzi G., Ruggenenti P. (2010). Predicting cisplatin-induced acute kidney injury by urinary neutrophil gelatinase-associated lipocalin excretion: A pilot prospective case-control study. Nephron Clin. Pract..

[21] Hirsch R., Dent C., Pfriem H., Allen J., Beekman R.H., Ma Q., Dastrala S., Bennett M., Mitsnefes M., Devarajan P. (2007). NGAL is an early predictive biomarker of contrast-induced nephropathy in children. Pediatr. Nephrol..

[22] Whiting P.H., Thomson A.W., Blair J.T., Simpson J.G. (1982). Experimental cyclosporin A nephrotoxicity. Br. J. Exp. Pathol..

[23] Murray B.M., Paller M.S., Ferris T.F. (1985). Effect of cyclosporine administration on renal hemodynamics in conscious rats. Kidney Int..

[24] Morgan C., Sis B., Pinsk M., Yiu V. (2011). Renal interstitial fibrosis in children treated with FK506 for nephrotic syndrome. Nephrol. Dial. Transplant..

[25] Smoter P., Nyckowski P., Grat M., Patkowski W., Zieniewicz K., Wronka K., Hinderer B., Morawski M. (2014). Risk factors of acute renal failure after orthotopic liver transplantation: Single-center experience. Transplant. Proc..

[26] Pageaux G.P., Rostaing L., Calmus Y., Duvoux C., Vanlemmens C., Hardgwissen J., Bernard P.H., Barbotte E., Vercambre L., Bismuth M. (2008). Mycophenolate mofetil in combination with reduction of calcineurin inhibitors for chronic renal dysfunction after liver transplantation. Liver Transplant..

[27] Neuberger J.M., Mamelok R.D., Neuhaus P., Pirenne J., Samuel D., Isoniemi H., Rostaing L., Rimola A., Marshall S., Mayer A.D. (2009). Delayed introduction of reduced-dose tacrolimus, and renal function in liver transplantation: The ‘ReSpECT’ study. Am. J. Transplant..

[28] Iwata H., Mizuno S., Ishikawa E., Tanemura A., Murata Y., Kuriyama N., Azumi Y., Kishiwada M., Usui M., Sakurai H. (2014). Negative prognostic impact of renal replacement therapy in adult living-donor liver transplant recipients: Preoperative recipient condition and donor factors. Transplant. Proc..

[29] Zhu F.X., Liu C.J., Zhu J.Y., Li G.M., Huang L., Wang D., Gao J., Zheng Z.M., Leng X.S. (2005). Risk factors of renal failure in the early post-liver transplantation period. Zhonghua Gan Zang Bing Za Zhi.

[30] Clauss A., Lilja H., Lundwall A. (2002). A locus on human chromosome 20 contains several genes expressing protease inhibitor domains with homology to whey acidic protein. Biochem. J..

[31] LeBleu V.S., Teng Y., O’Connell J.T., Charytan D., Muller G.A., Muller C.A., Sugimoto H., Kalluri R. (2013). Identification of human epididymis protein-4 as a fibroblast-derived mediator of fibrosis. Nat. Med..

[32] Yang Z., Zhang Z., Qin B., Wu P., Zhong R., Zhou L., Liang Y. (2016). Human Epididymis Protein 4: A Novel Biomarker for Lupus Nephritis and Chronic Kidney Disease in Systemic Lupus Erythematosus. J. Clin. Lab. Anal..

[33] Luo J., Wang F., Wan J., Ye Z., Huang C., Cai Y., Liu M., Wu B.Q., Li L. (2018). Serum human epididymis secretory protein 4 as a potential biomarker of renal fibrosis in kidney transplantation recipients. Clin. Chim. Acta.

[34] Nakagawa S., Nishihara K., Miyata H., Shinke H., Tomita E., Kajiwara M., Matsubara T., Iehara N., Igarashi Y., Yamada H. (2015). Molecular Markers of Tubulointerstitial Fibrosis and Tubular Cell Damage in Patients with Chronic Kidney Disease. PLoS ONE.

