# Supplementary material for: Neutrophil Gelatinase-Associated Lipocalin Is Not Associated with Tacrolimus-Induced Acute Kidney Injury in Liver Transplant Patients Who Received Mycophenolate Mofetil with Delayed Introduction of Tacrolimus
Source: Int J Mol Sci. 2019 Jun 25;20(12):3103. doi: 10.3390/ijms20123103 (PMC6627315; doi:10.3390/ijms20123103)
Supplement: Supplementary file 1 [file ijms-20-03103-s001.pdf]

Supplementary Material:

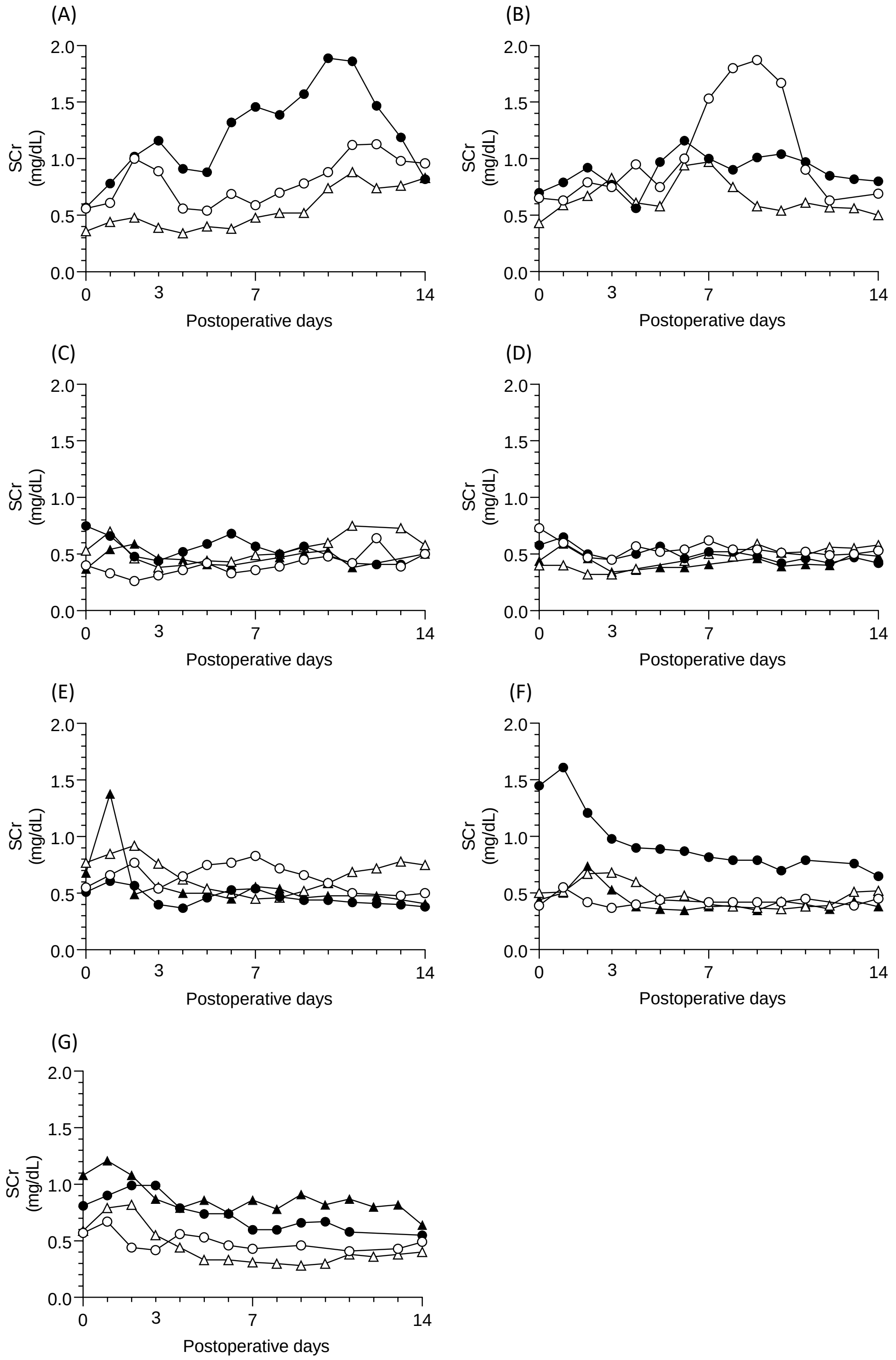

Figure S1. Time-dependent changes in SCr levels in the liver transplant patients who experienced tacrolimus-induced AKI (**A**,**B**) and non-AKI patients (**C–G**) during the period of Postoperative Days 0–14 are summarized. Elevation of SCr within three postoperative days was diagnosed as tacrolimus-unrelated AKI by the physicians because the induction of tacrolimus was set on Postoperative Day 2 or after.
